# Supplementary material for: MDMX Regulates Transcriptional Activity of p53 and FOXO Proteins to Stimulate Proliferation of Melanoma Cells
Source: Cancers (Basel). 2022 Sep 15;14(18):4482. doi: 10.3390/cancers14184482 (PMC9496676; doi:10.3390/cancers14184482)
Supplement: Supplementary file 1 [file cancers-14-04482-s001.zip › Supplementary figures.pdf]

Supplementary Figure S1

A

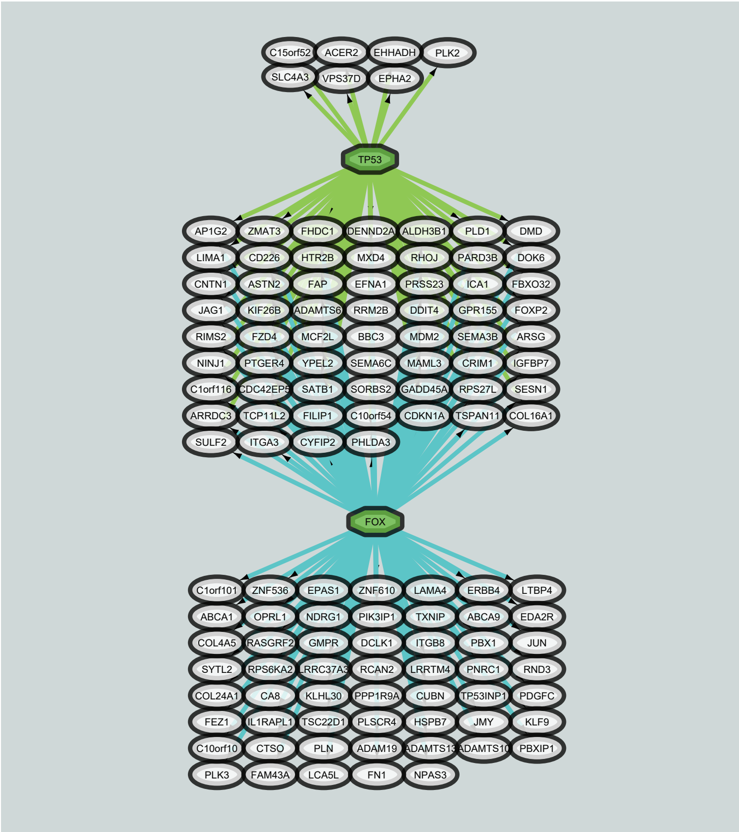

B

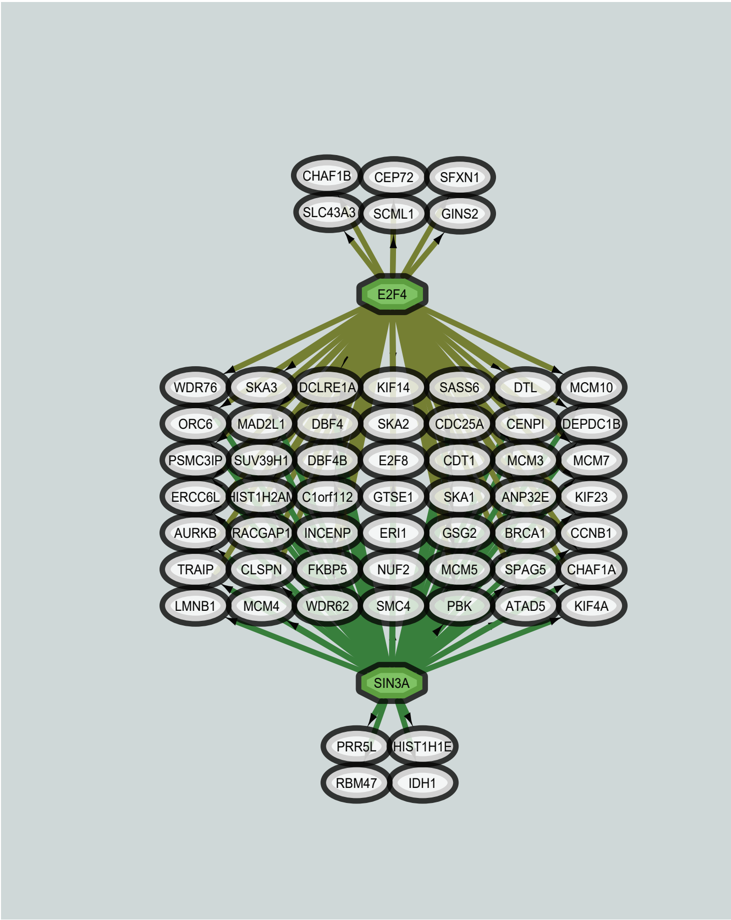

Supplementary figure S1. Gene regulatory network of genes transcriptionally affected upon MDMX knockdown.

A) Gene regulatory network of genes upregulated upon MDMX depletion identifying the two major regulators p53 and Forkhead box (FOX) transcription regulators

B) Gene regulatory network of genes downregulated upon MDMX depletion identifying the two major regulators E2F4 and SIN3A.

Supplementary Figure S2

A

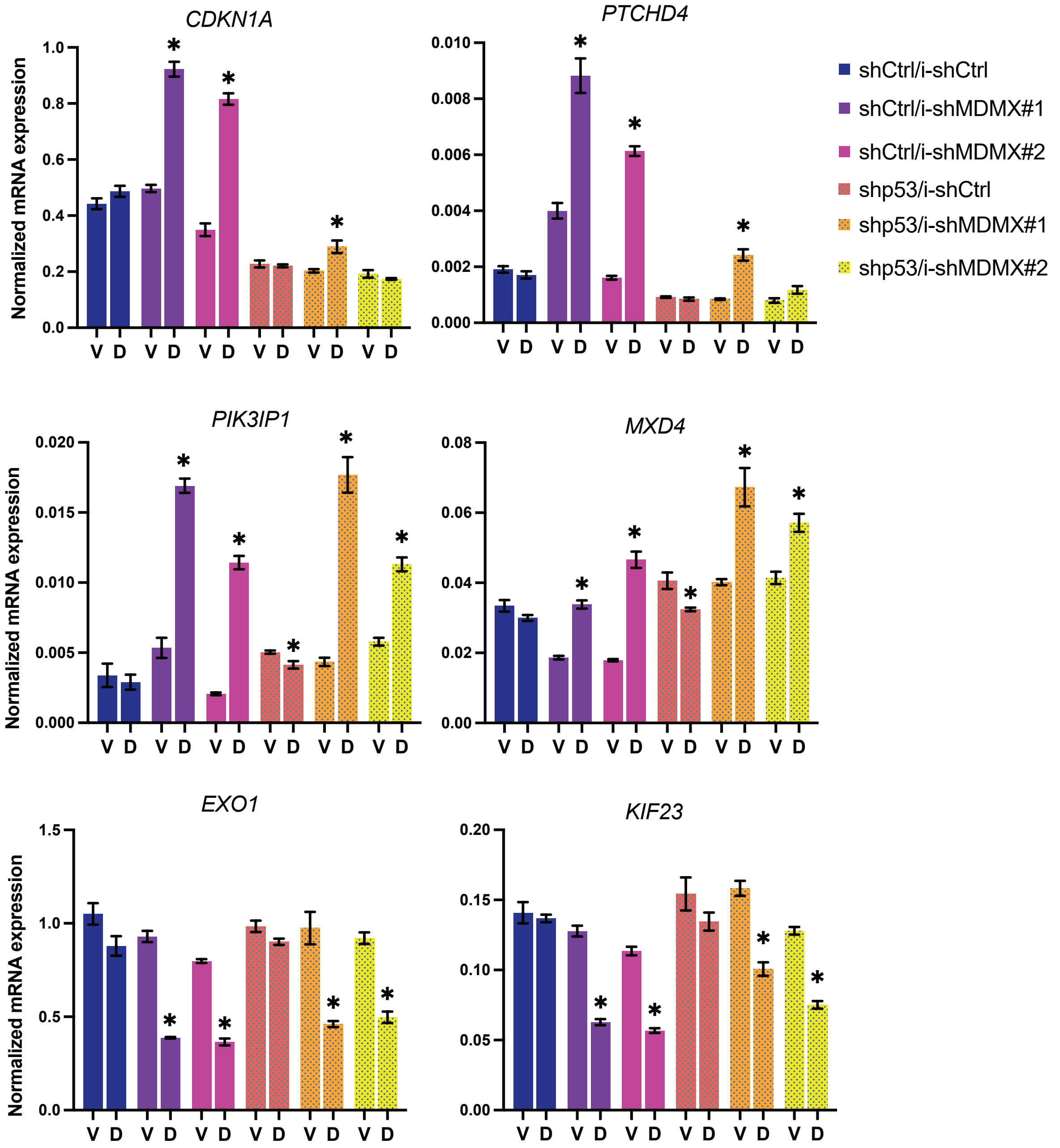

B

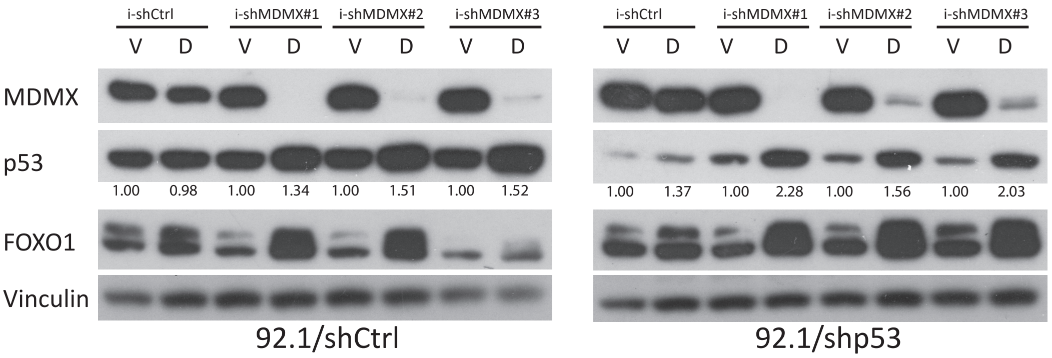

Supplementary figure S2. Transcriptional effects upon MDMX depletion in 92.1 cells.
A) Normalized mRNA expression of the upregulated genes *CDKN1A, PTCHD4, PIK3IP1* and *MXD4* and of the downregulated genes *EXO1* and *KIF23*, upon MDMX depletion in 92.1/shCtrl and 92.1/shp53 cells. Cells were treated with doxycycline (D; 10 ng/ml) or vehicle (V) for 48 hrs. Significant alterations ($p < 0.05$) in expression levels are indicated with *.
B) Analysis of protein expression upon MDMX depletion in 92.1/shCtrl and 92.1/shp53 cells.

Supplementary Figure S3

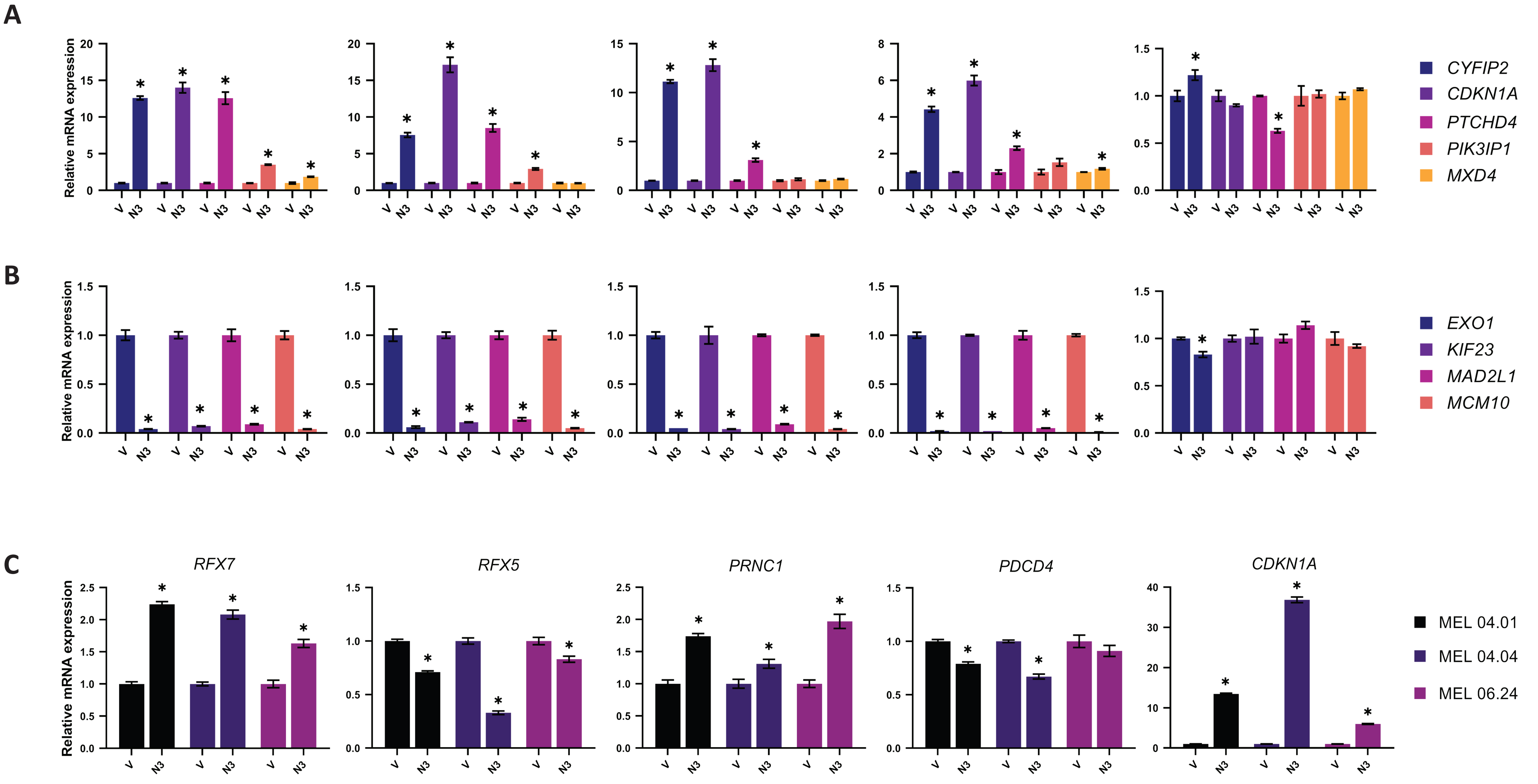

Supplementary figure S3. Effects of Nutlin-3 treatment on expression of 'MDMX target genes' in uveal and cutaneous melanoma cell lines. Normalized, relative mRNA expression of the upregulated MDMX target genes *CYFIP2, CDKN1A, PTCHD4, PIK3IP1* and *MXD4* (A), and the downregulated MDMX target genes *EXO1, KIF23, MAD2L1, MCM10* (B) upon 24 hours of Nutlin-3 treatment (N3; 10 µM) or Vehicle (V) in uveal melanoma cell lines MEL202 and 92.1 and cutaneous melanoma cell lines MEL 04.01, MEL 06.24 and MEL 94.07.

C) Normalized mRNA expression of *RFX7* and the RFX7-target genes *RFX5, PNRC1* and *PDCD4* upon 24 hrs Nutlin-3 treatment (N3; 10 µM) or Vehicle (V) in the cutaneous melanoma cell lines MEL 04.01, MEL 04.04 and MEL 06.24. Expression of the classical p53 target gene *CDKN1A* was analyzed as positive control.

Significant alterations ($p < 0.05$) in expression levels are indicated with *.

Supplementary Figure S4

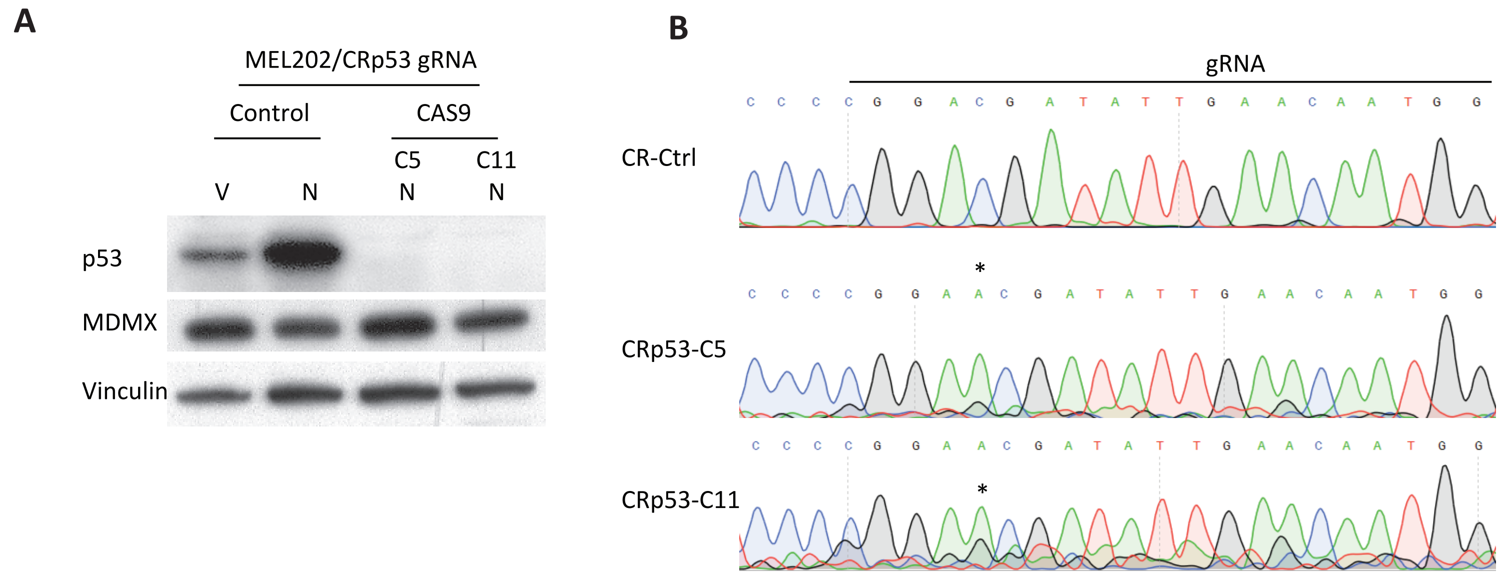

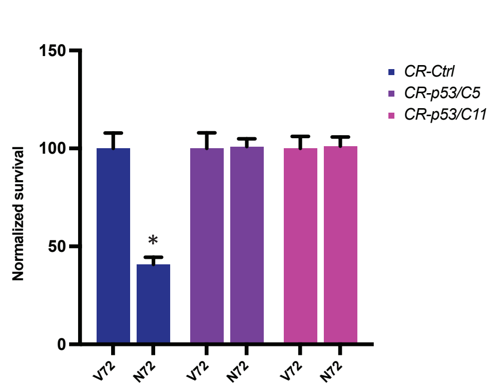

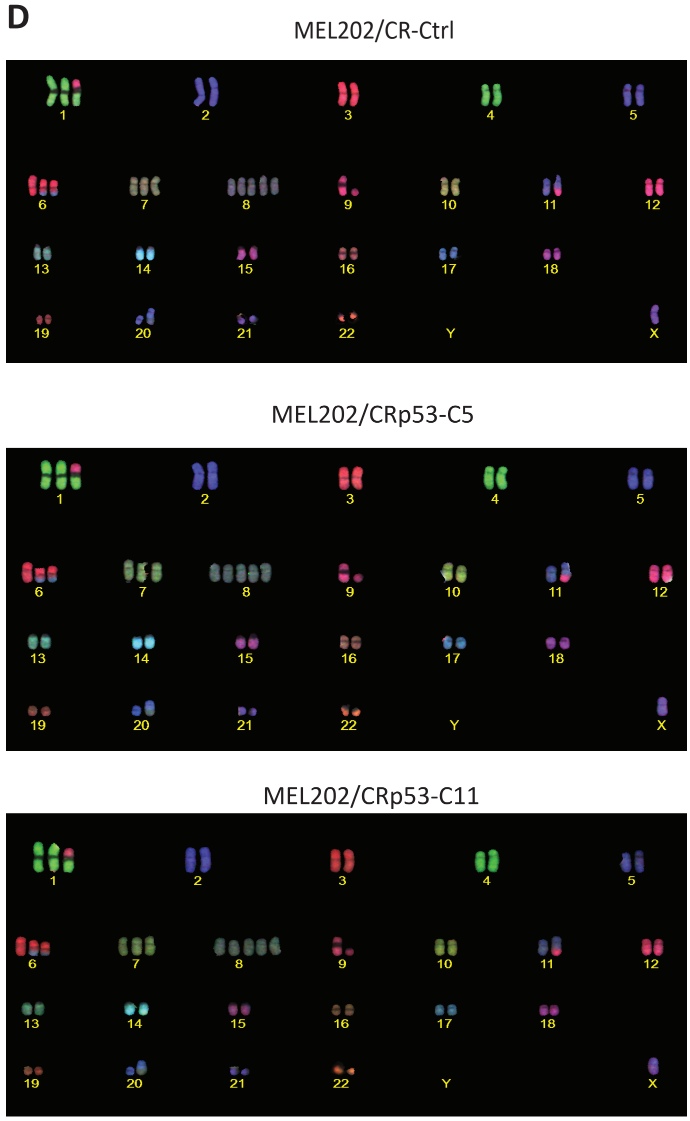

Supplementary figure S4.
Generation of p53 knock-out MEL202 cells.
A) Analysis of protein expression of MEL202 control cells and two MEL202-derived p53 knockout cells (CRp53/C5 and CRp53/C11) treated for 24 hrs with DMSO (D) or 4 µM Nutlin-3 (N). The p53 protein levels increased in the control cells upon Nutlin-3 treatment, but remained undetectable in the p53 knockout cells. MDMX levels showed minor differences between clones.
B) Sanger sequencing of control and p53 knockout clones. Shown are the guide RNA sequence and the insertion of one adenine, highlighted by the asterisk (*).
C) Relative survival of MEL202/CR-Ctrl and MEL202/CRp53 cells upon 4 µM Nutlin-3 treatment for 72 hrs. Significant alterations ($p < 0.05$) are indicated with *.
D) COBRA-FISH analysis of chromosomal content of MEL202/CR-Ctrl control and two p53-KO cell lines, CRp53/C5 and CRp53/C11

Supplementary Figure S5

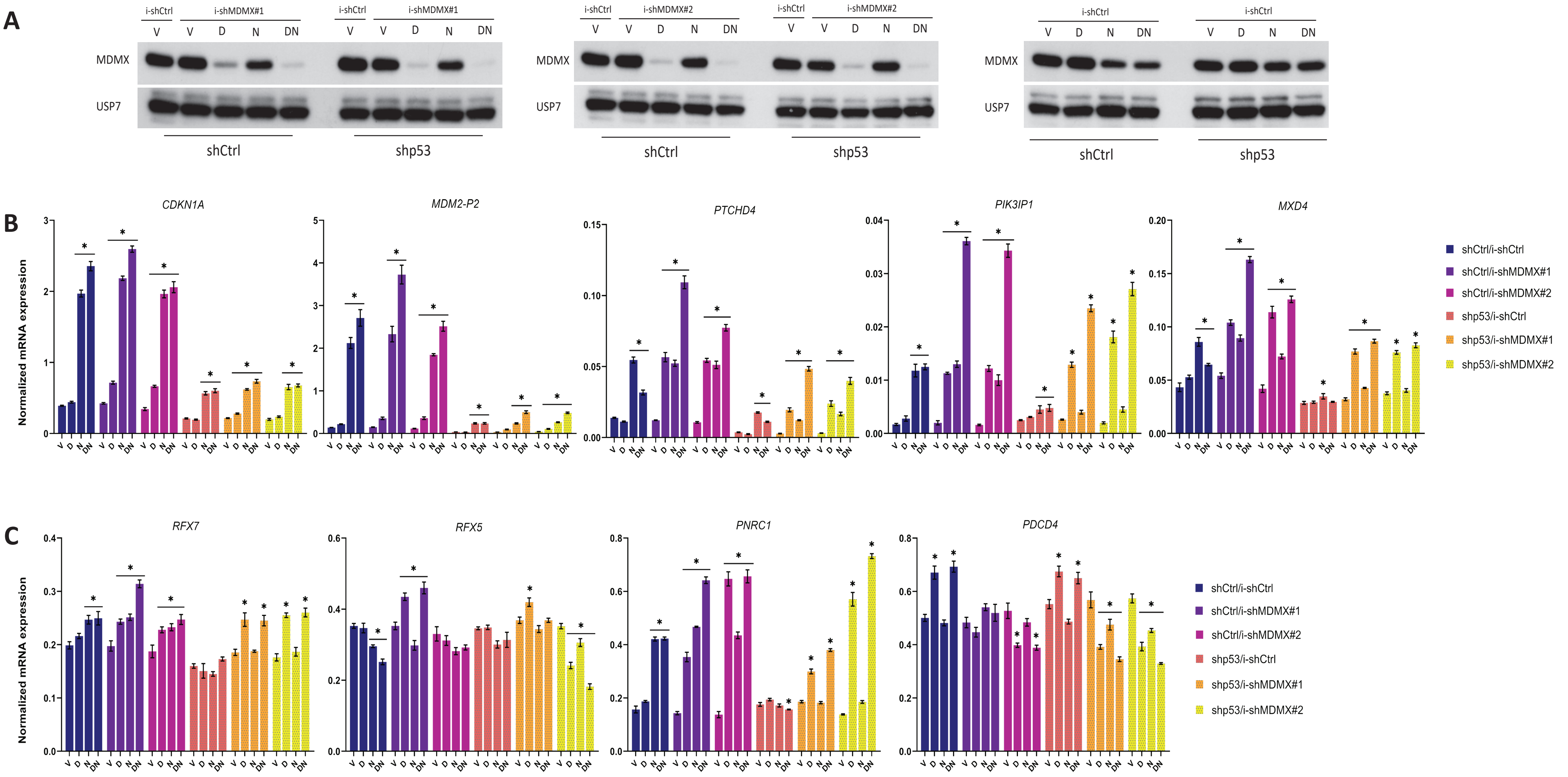

Supplementary figure S5. Effects of MDMX depletion in MEL 93.05 cutaneous melanoma cell line.

Indicated cell lines were treated with Vehicle (V) or doxycycline (D; 10 ng/ml), in the absence or presence of Nutlin-3 (N; 10 μM) for 72 hrs after which RNA and protein were harvested.

A) Analyses of protein lysates for the efficiency of MDMX depletion. USP7 was analysed to show equal loading.

B) Analysis of mRNA expression of the p53-target genes *CDKN1A, MDM2* and *PTCHD4*, and the FOX(O) target genes *PIK3IP1* and *MXD4*.

C) Analysis of mRNA expression of *RFX7* and reported RFX7-target genes *RFX5, PNRC1* and *PDCD4*.

Significant alterations ($p < 0.05$) in expression levels are indicated with *.

Supplementary Figure S6

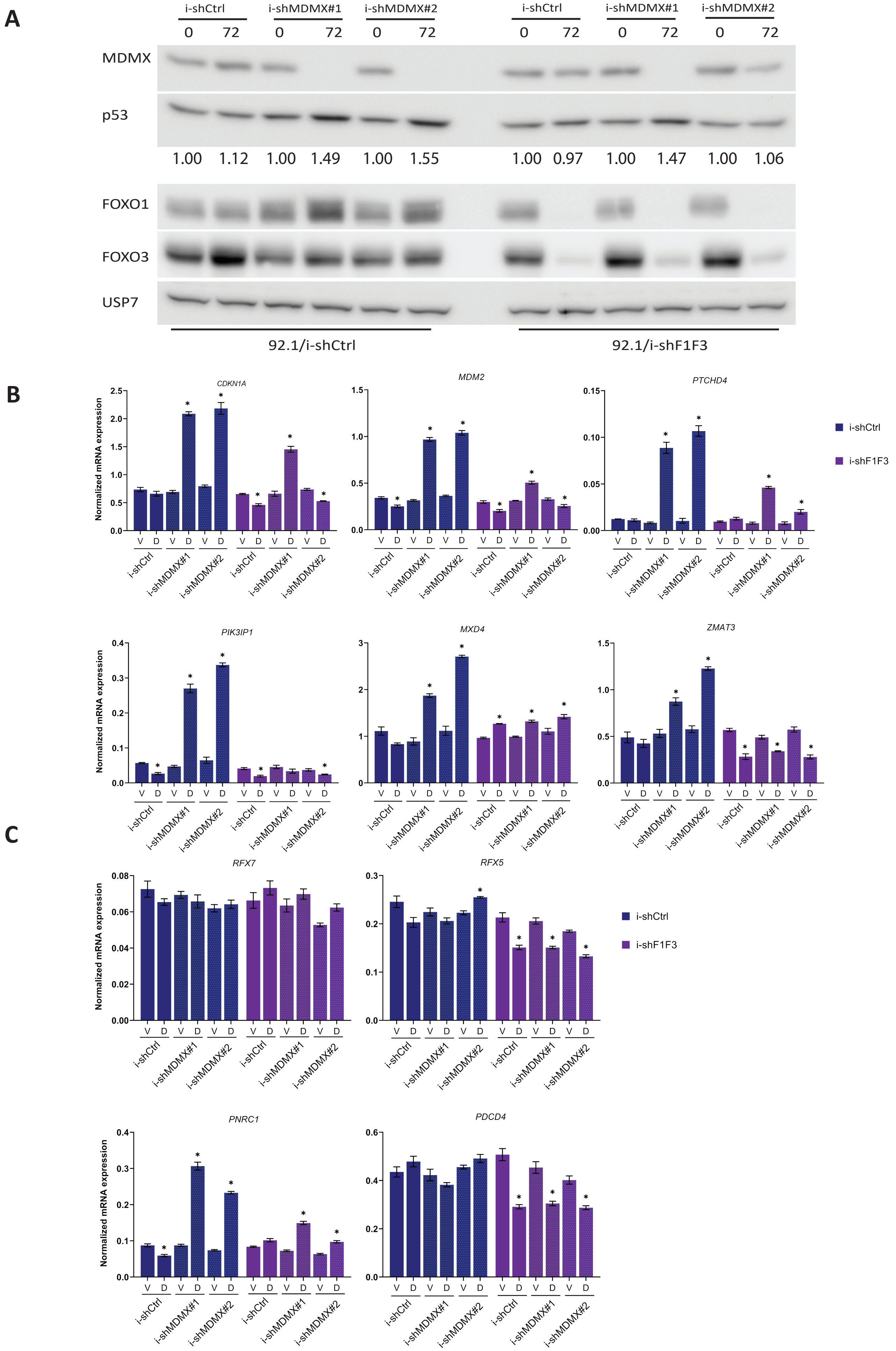

Supplementary figure S6. Effect of MDMX depletion in the uveal melanoma cell line 92.1, shCtrl and shp53. Indicated cell lines were treated with doxycycline (10 ng/ml) for 72 hrs after which RNA and protein were harvested. A) Protein lysates were analysed by Western blotting to assess the efficacy of depletion of MDMX, FOXO1, FOXO3, and the effect on p53 protein levels. USP7 was analysed to show equal loading. B) Analysis of mRNA expression of the p53-target genes *CDKN1A, MDM2* and *PTCHD4*, the FOX(O) target genes *PIK3IP1* and *MXD4* and the dual p53/FOXO target gene *ZMAT3*. C) Analysis of mRNA expression of *RFX7* and the reported RFX7-target genes *RFX5, PNRC1* and *PDCD4*. Significant alterations ($p < 0.05$) in expression levels are indicated with *.

Supplementary Figure S7

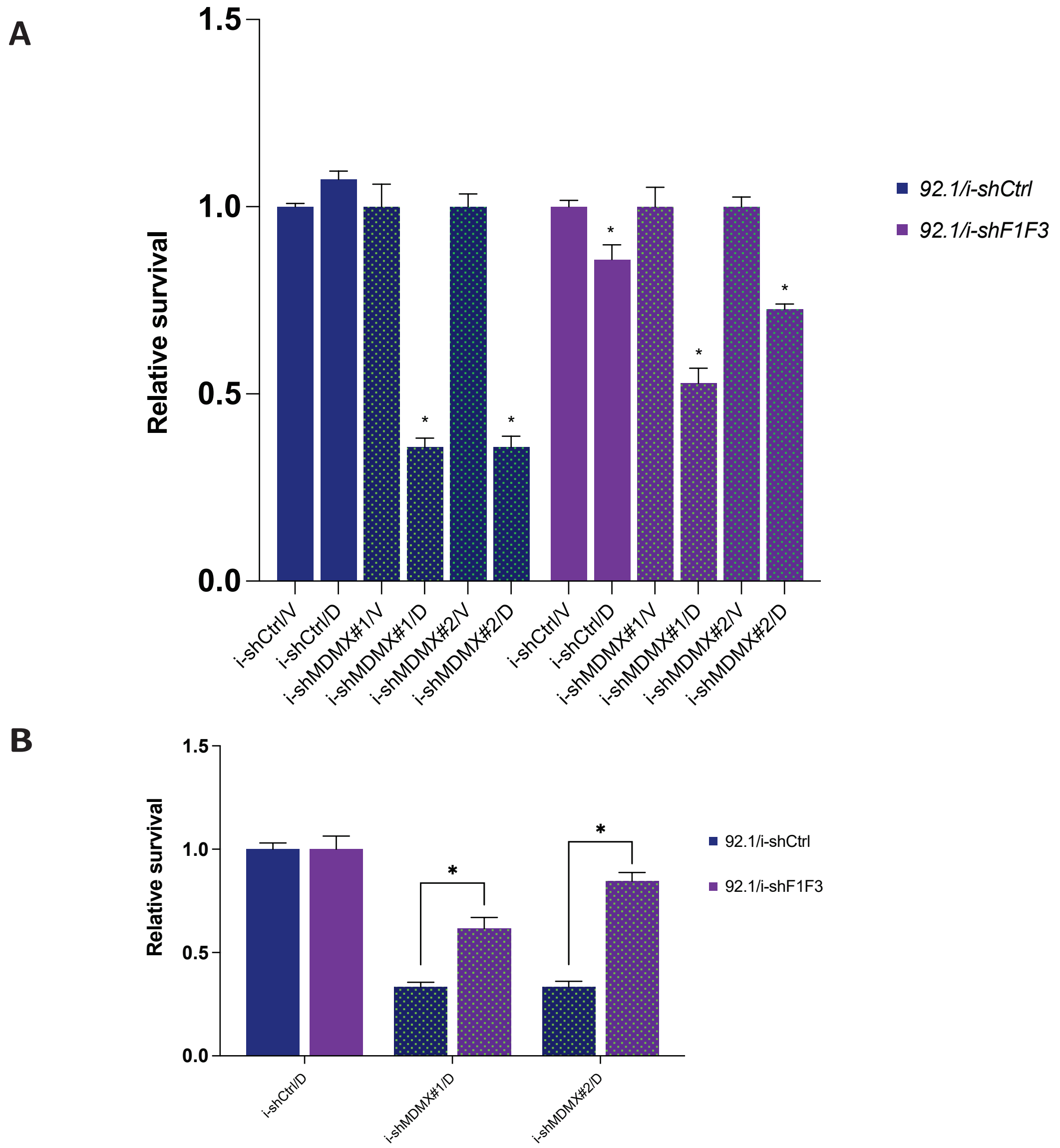

Supplementary Figure S7. MDMX stimulates the growth of UM cell line 92.1 via attenuation of p53 and FOXO activity.

A) The 92.1-derived cell lines (i-shCtrl or i-shFOXO1/FOXO3), either containing an i-shCtrl or two distinct i-shMDMX shRNA constructs, were treated with doxycycline (D; 10 ng/ml) for 72 hrs. Relative survival of the cell lines, each normalized to Vehicle (V)-treated samples.

B) Relative survival of the indicated cell lines but now normalized to i-shCtrl+Doxycycline to determine the effect of MDMX depletion in i-shCtrl versus i-shFOXO cells.

Significant alterations ($p < 0.05$) are indicated with *.
