# Supplementary material for: MDMX Regulates Transcriptional Activity of p53 and FOXO Proteins to Stimulate Proliferation of Melanoma Cells
Source: Cancers (Basel). 2022 Sep 15;14(18):4482. doi: 10.3390/cancers14184482 (PMC9496676; doi:10.3390/cancers14184482)

Blots Figure 1A

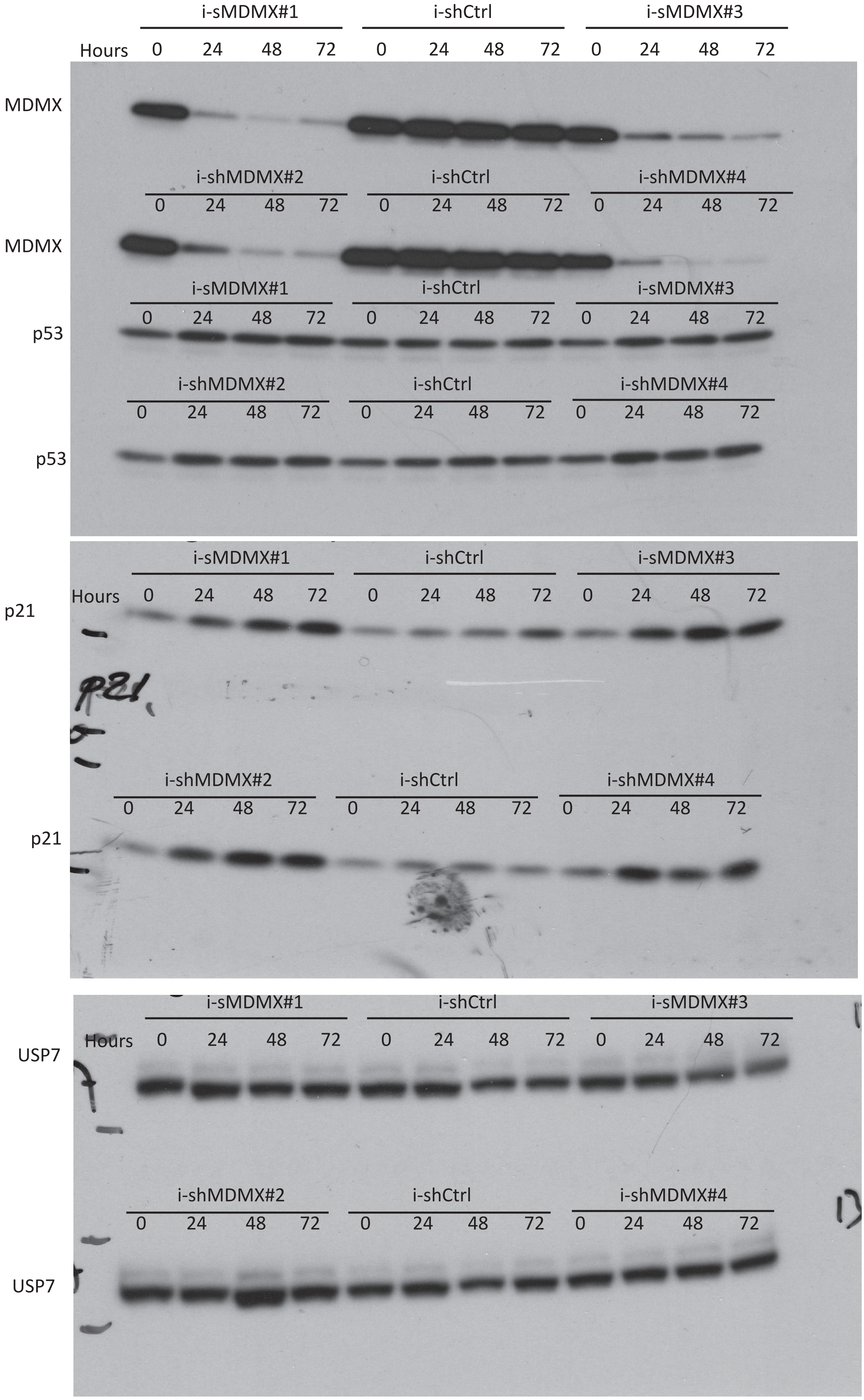

Blots Figure 2B

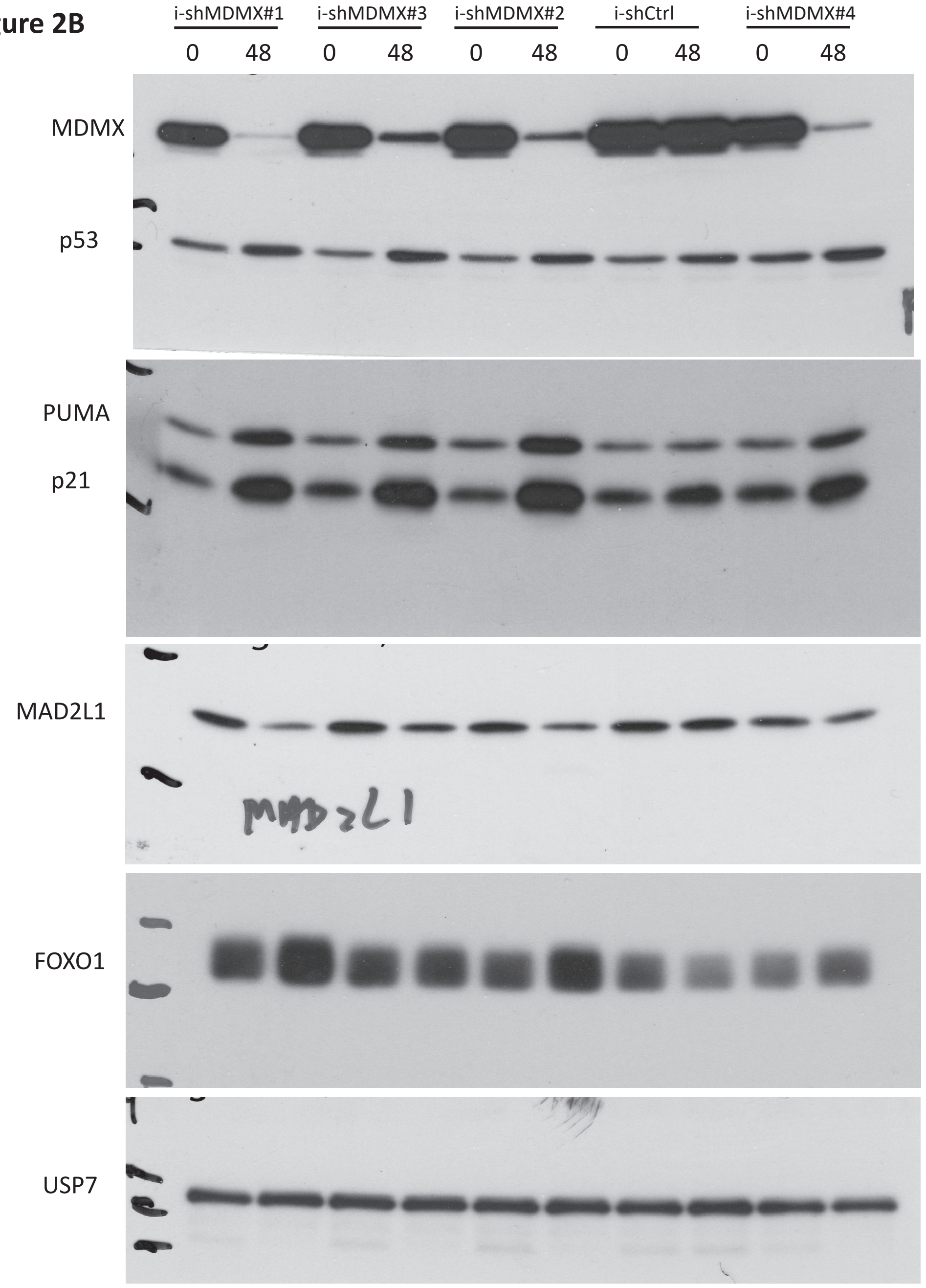

Blots Figure 2D

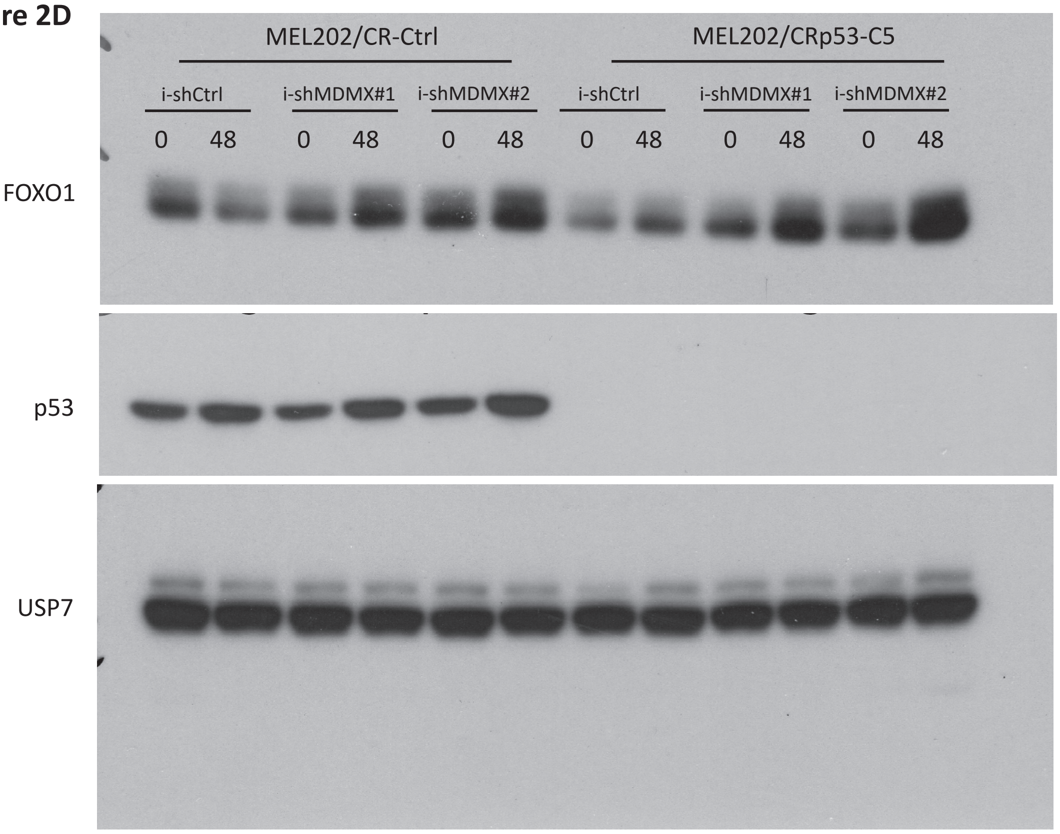

Blots Figure 3A

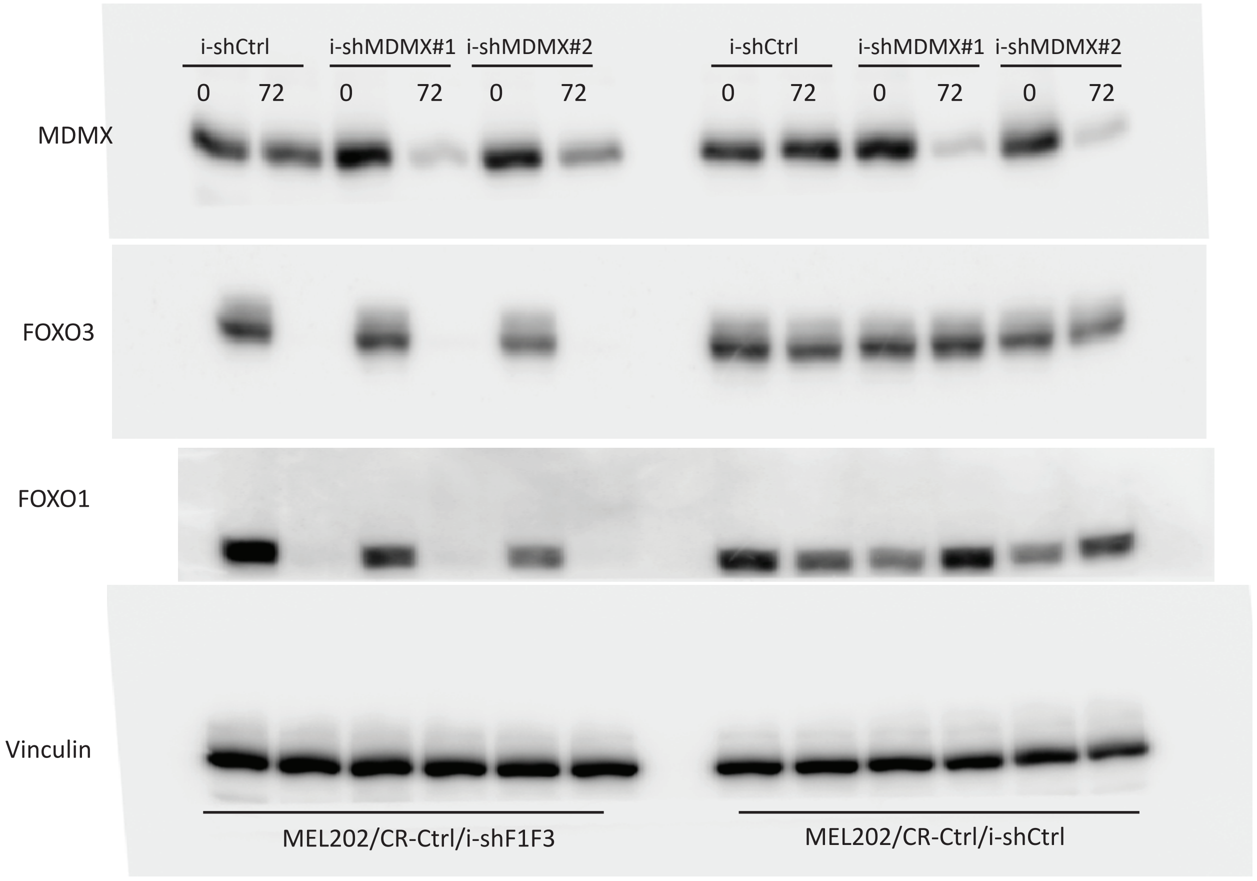

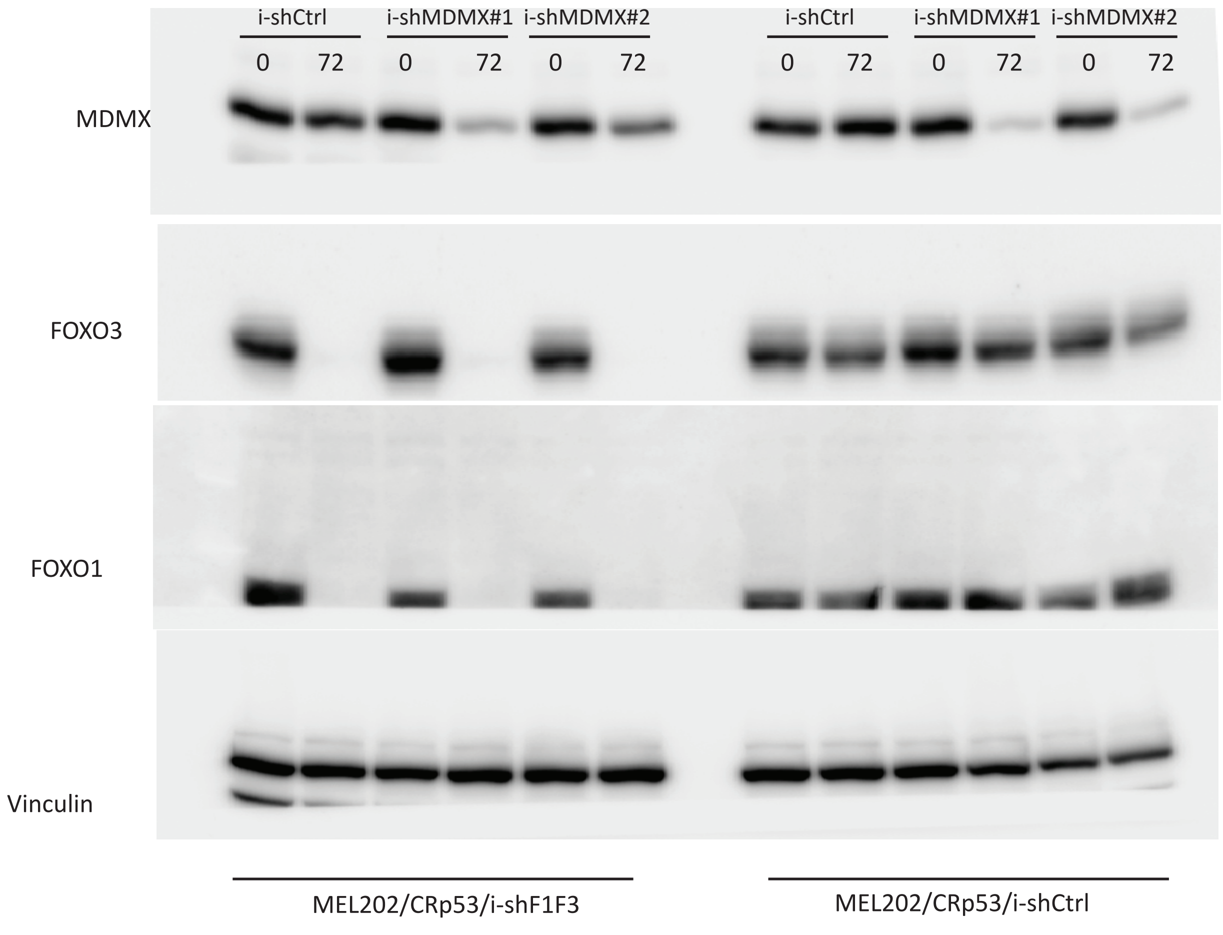

Blots Figure 5B

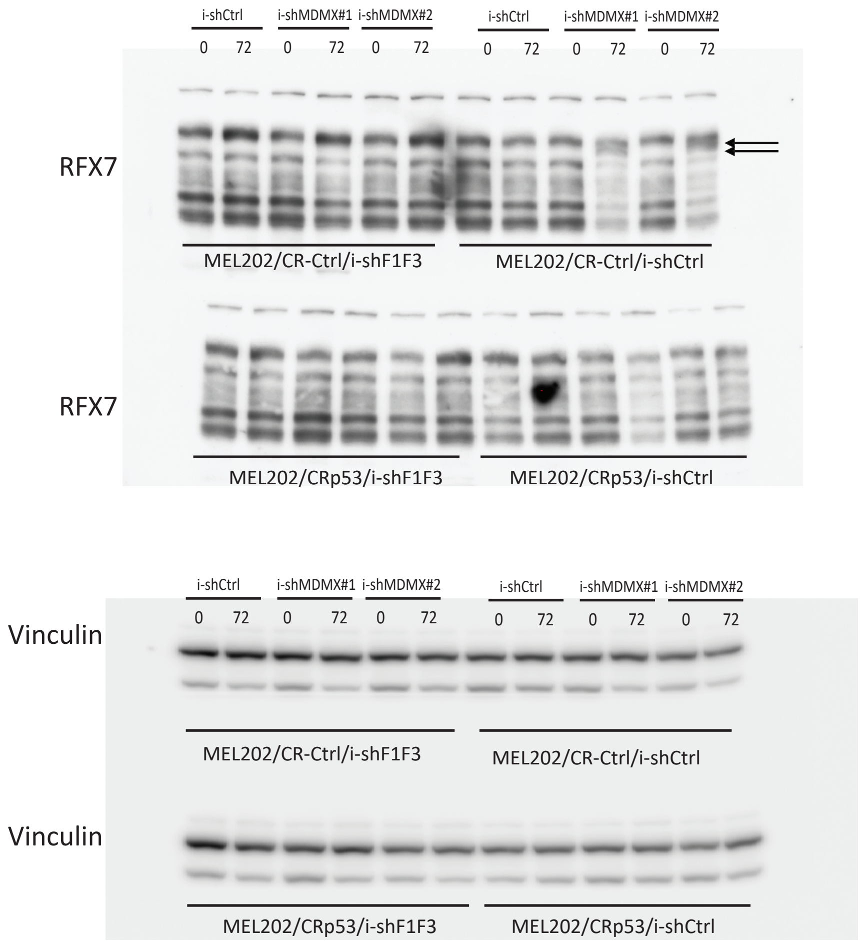

Blots Figure 5C

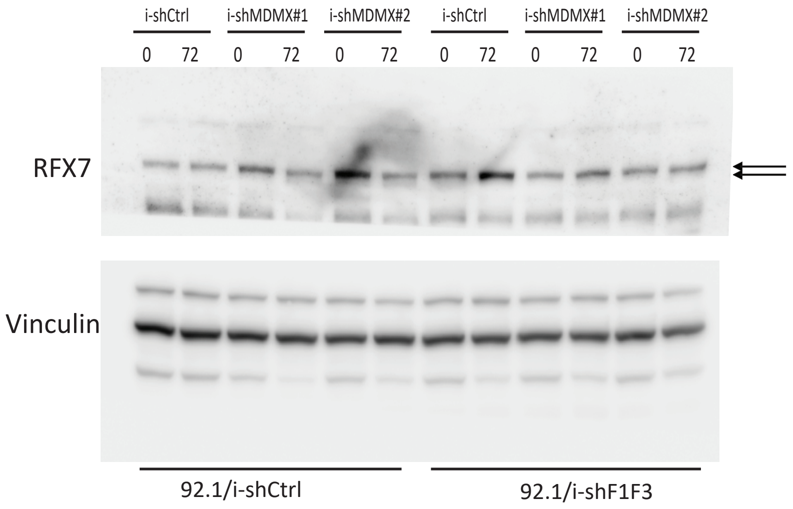

Blots Figure S2 B

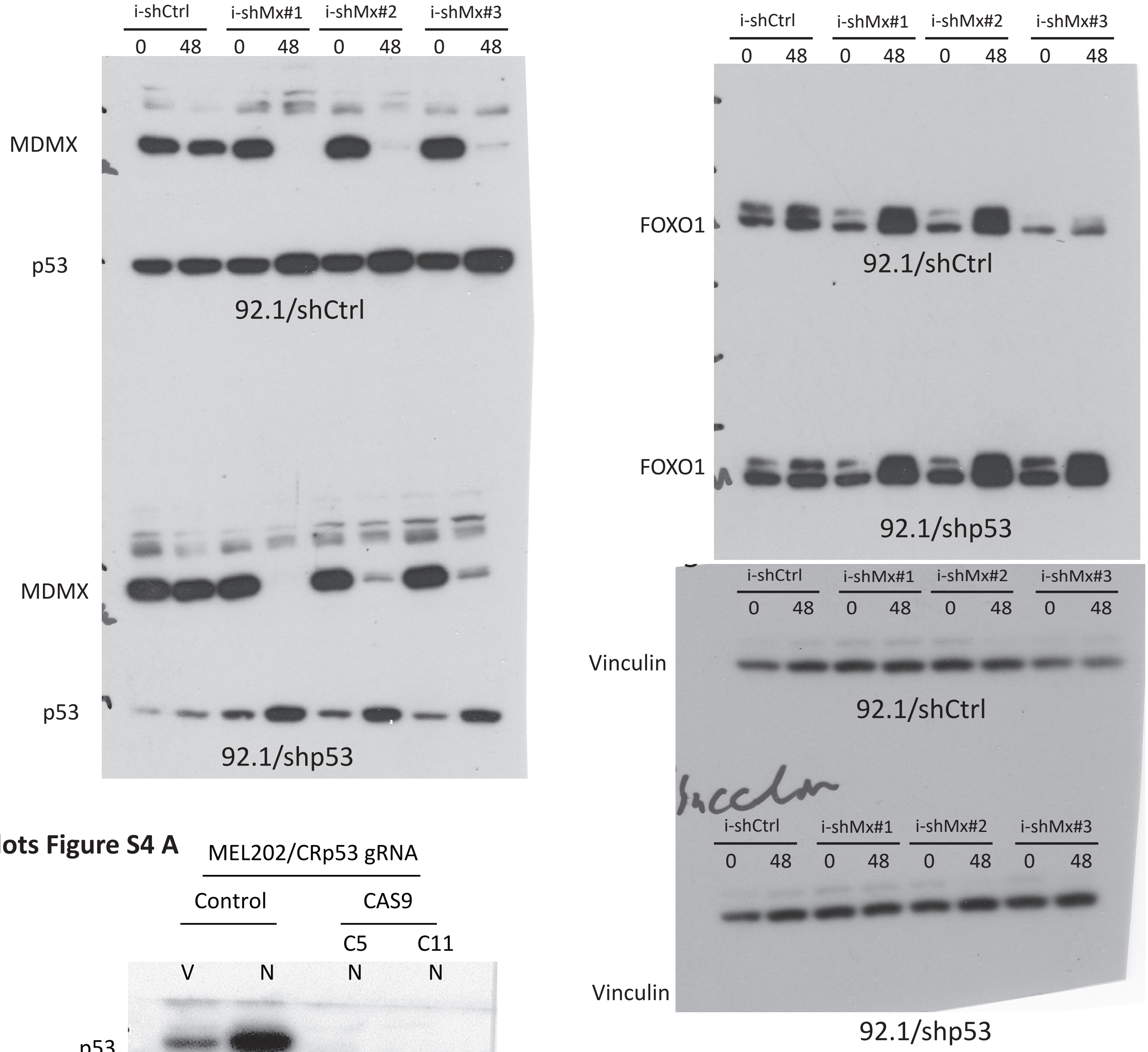

Blots Figure S4 A

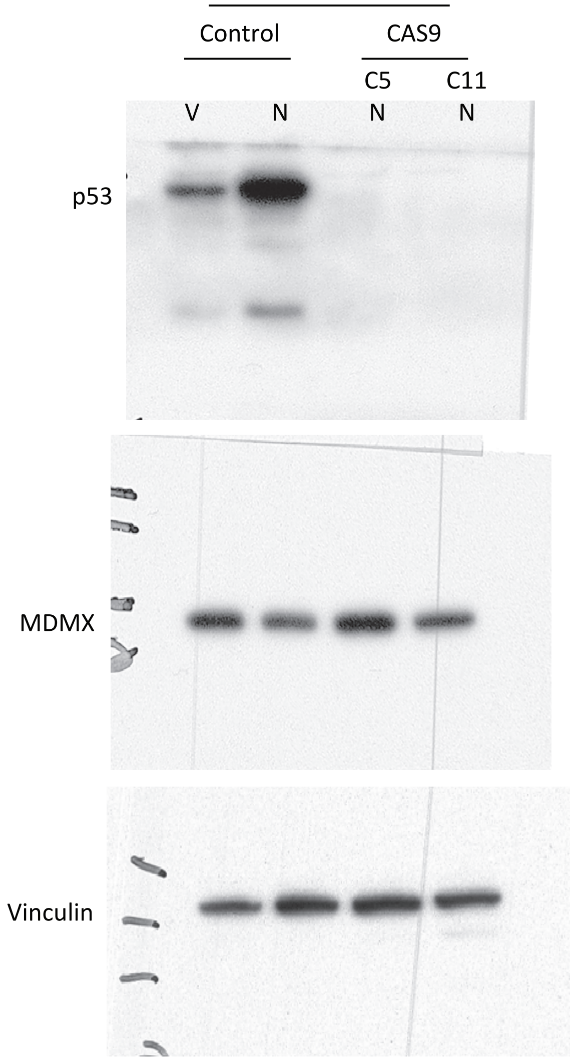

Blots Figure S5 A

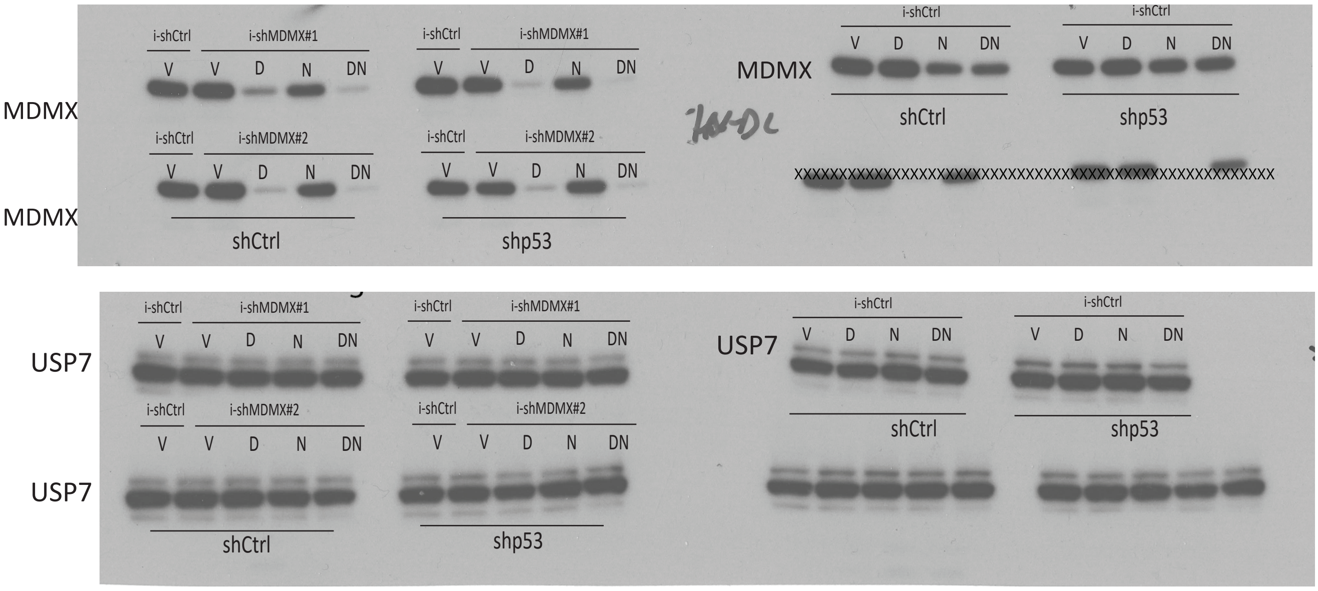

Blots Figure S6 A

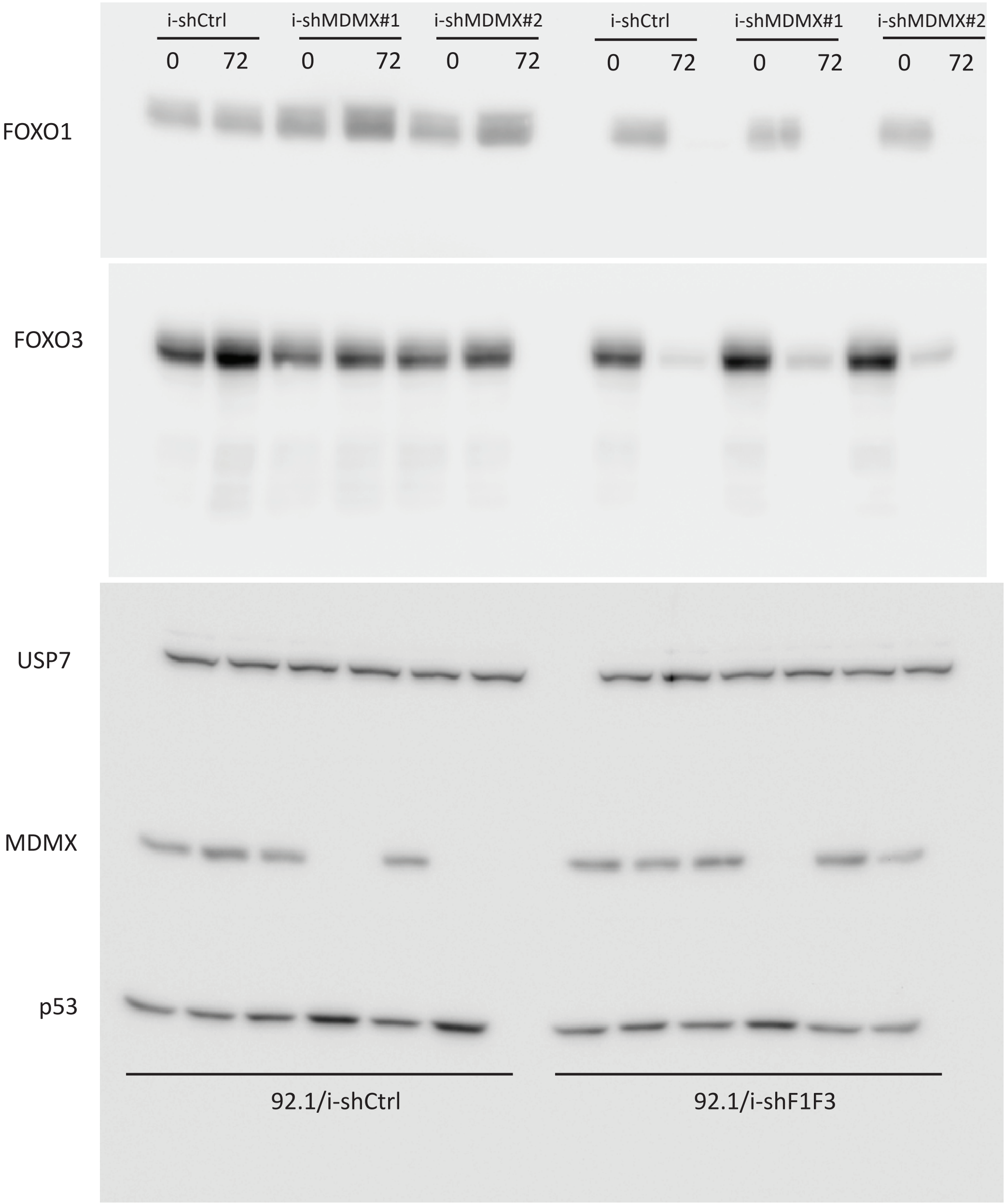

Supplement: Supplementary file 1 [file cancers-14-04482-s001.zip › Supplementary file S1.pdf]
